# Supplementary material for: Oxygen Vacancy-Enhanced Ni3N-CeO2/NF Nanoparticle Catalysts for Efficient and Stable Electrolytic Water Splitting
Source: Nanomaterials (Basel). 2024 May 26;14(11):935. doi: 10.3390/nano14110935 (PMC11173528; doi:10.3390/nano14110935)
Supplement: Supplementary file 1 [file nanomaterials-14-00935-s001.zip › nanomaterials-3008052-supplementary.pdf]

Oxygen Vacancy-Enhanced Ni_3N-CeO_2/NF Nanoparticle Catalysts for Efficient and Stable Electrolytic Water Splitting

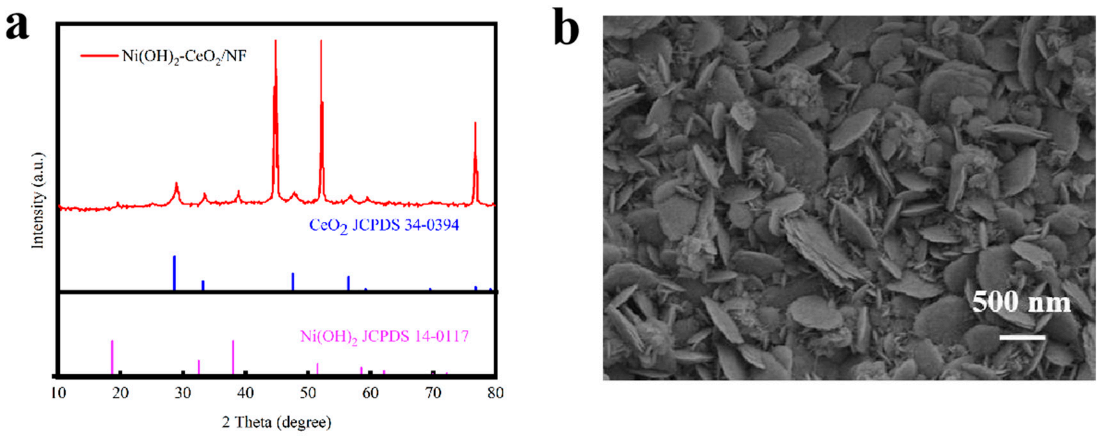

Figure S1. XRD of Ni(OH)$_2$-CeO$_2$/NF and SEM image of Ni(OH)$_2$-CeO$_2$/NF.

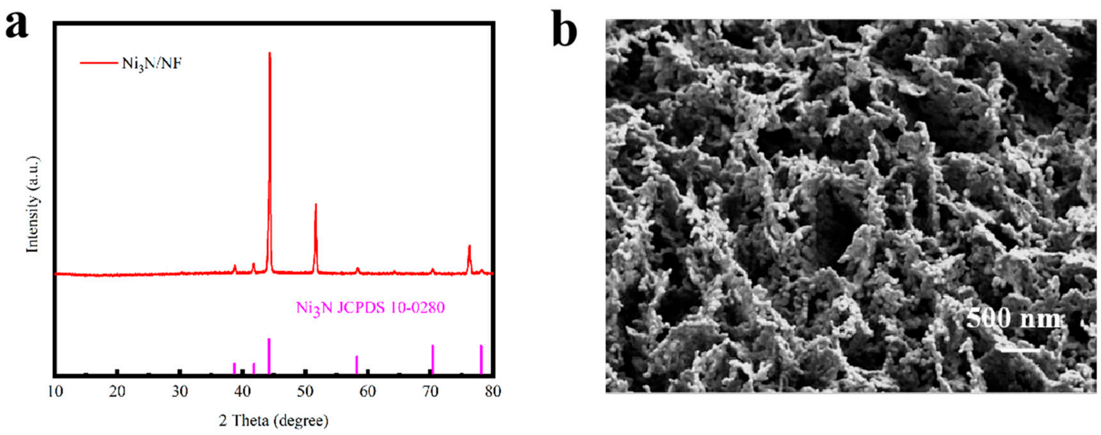

Figure S2. XRD of Ni$_3$N/NF and SEM image of Ni$_3$N/NF.

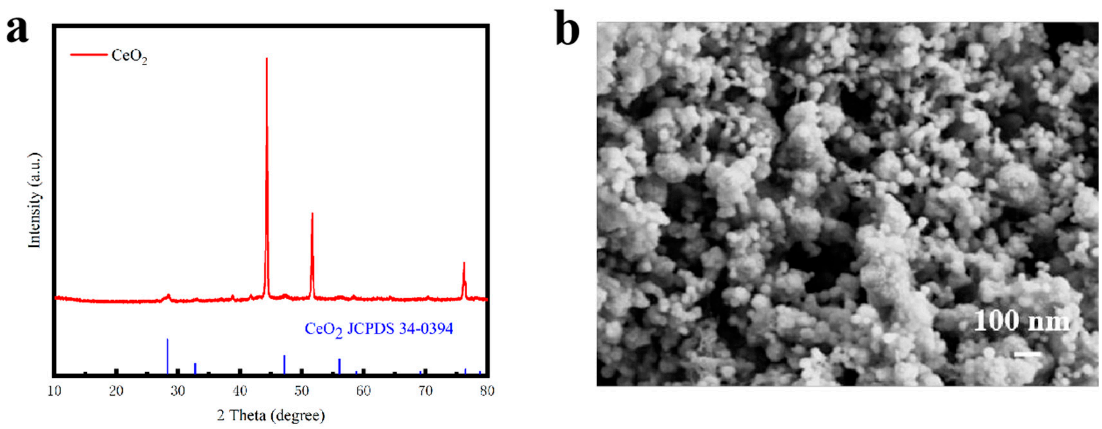

Figure S3. XRD of CeO$_2$ and SEM image of CeO$_2$.

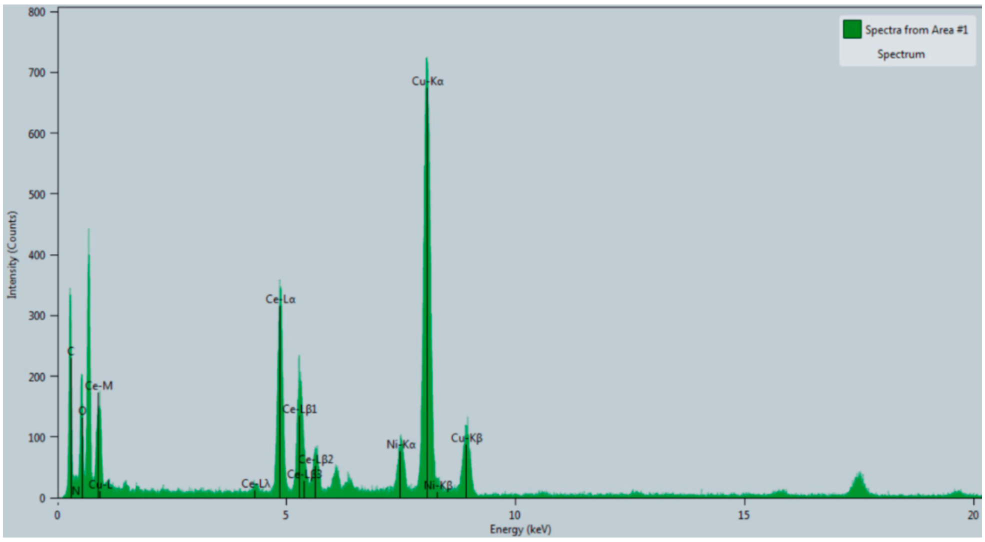

Figure S4. a) The corresponding energy dispersive Xtray (EDX) images of the Ni_3N-CeO_2/NF.

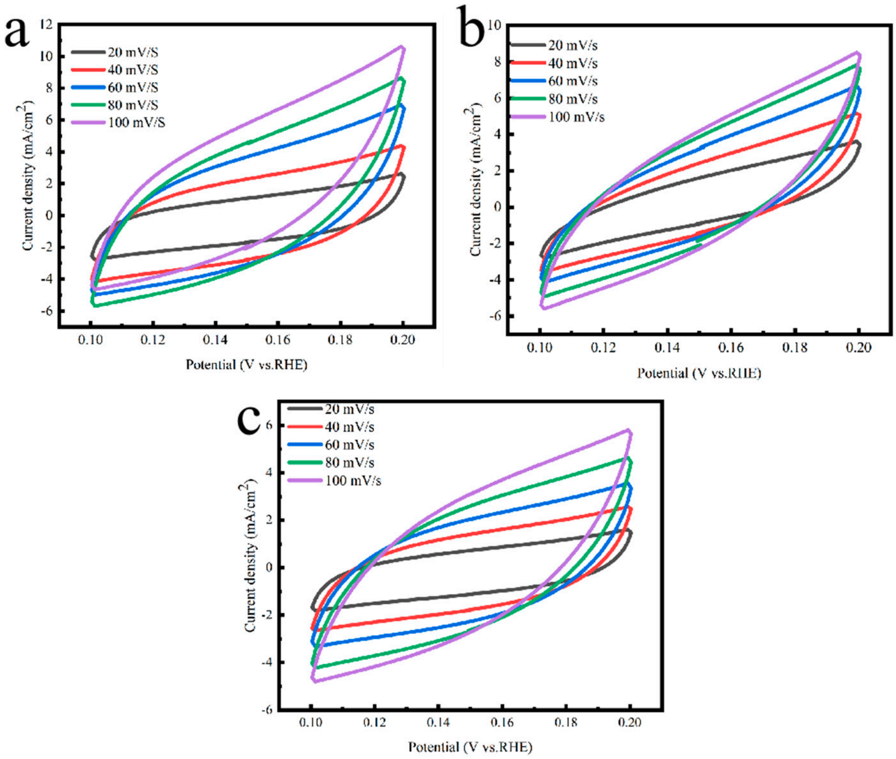

Figure S5. CV curves of at 0.125-0.225 V vs RHE with the non-Faradaic potential regions from 20-100 mV/s for (a) Ni_3N-CeO_2/NF, (b) Ni_3N/NF, and (c) CeO_2/NF.

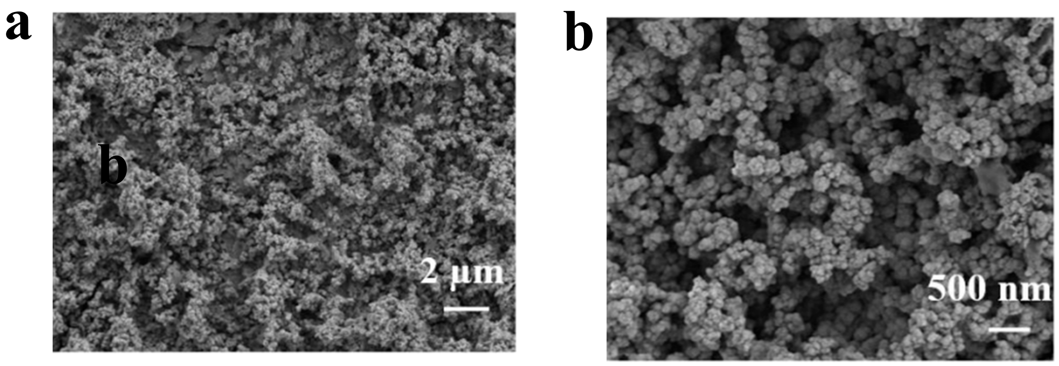

Figure S6. SEM images of Ni_3N-CeO_2/NF after HER stability test.

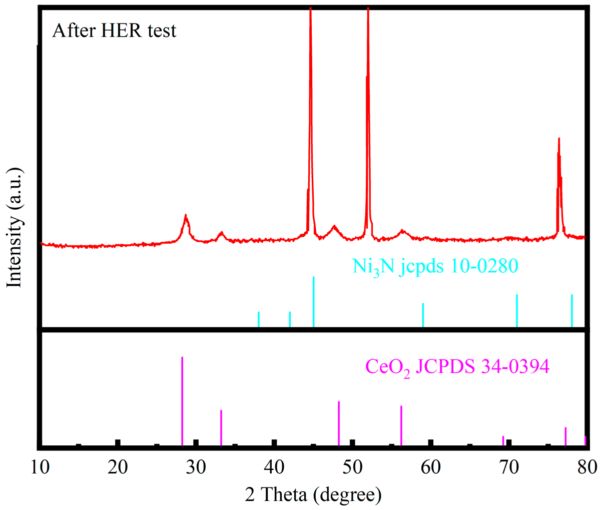

Figure S7. XRD images of Ni_3N-CeO_2/NF after HER stability test.

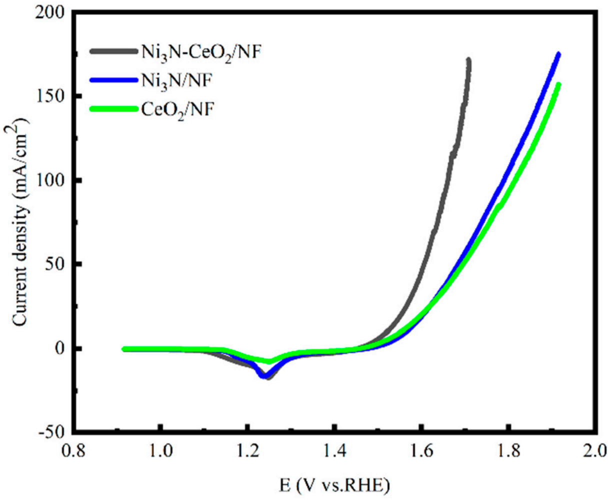

Figure S8. Backscatter polarization curves of OER for Ni_3N-CeO_2/NF, Ni_3N/NF and CeO_2/NF.

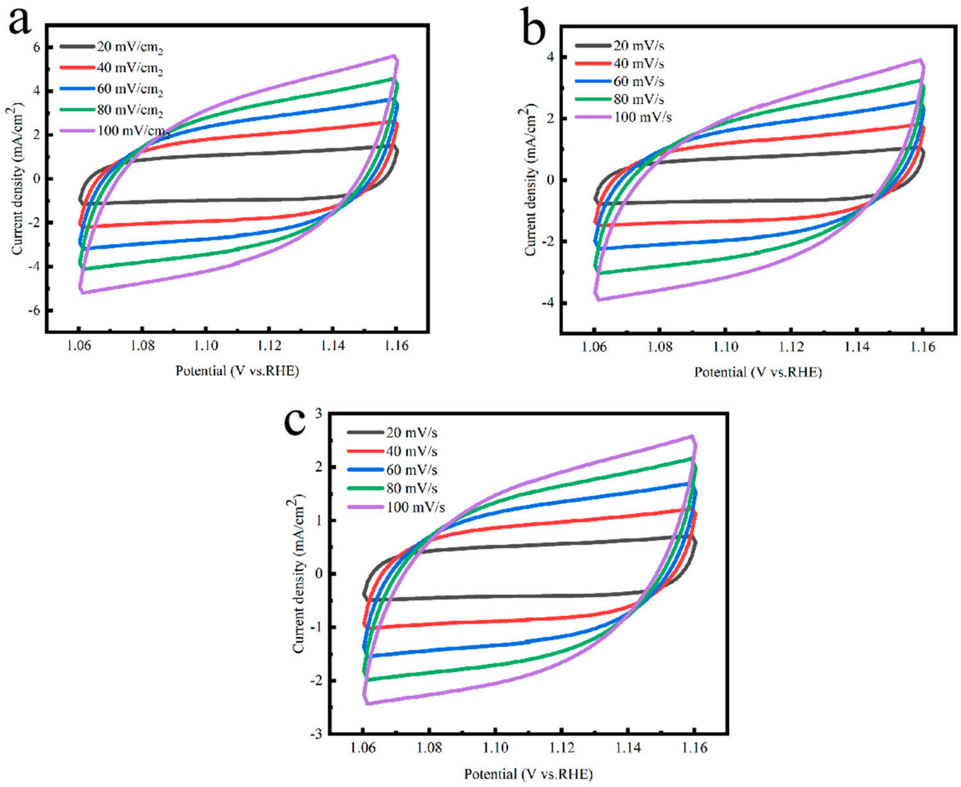

Figure S9. CV curves of at 0.125-0.225 V vs RHE with the non-Faradaic potential regions from 20-100 mV/s for (a) Ni_3N-CeO_2/NF, (b) Ni_3N/NF, and (c) CeO_2/NF.

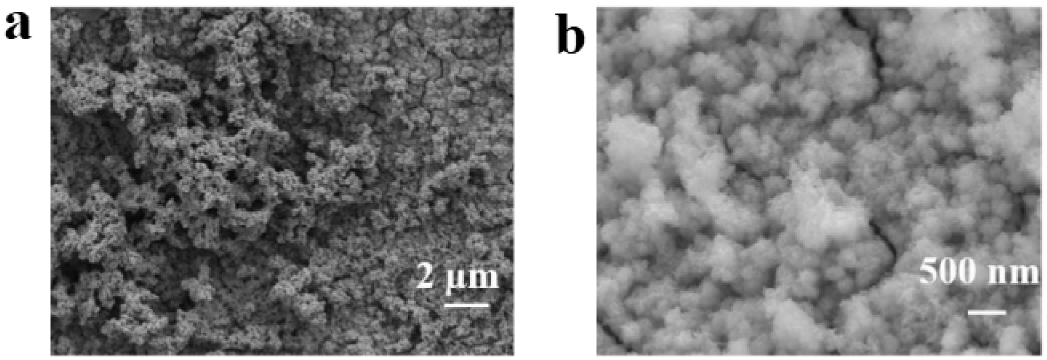

Figure S10. SEM images of Ni_3N-CeO_2/NF after HER stability test.

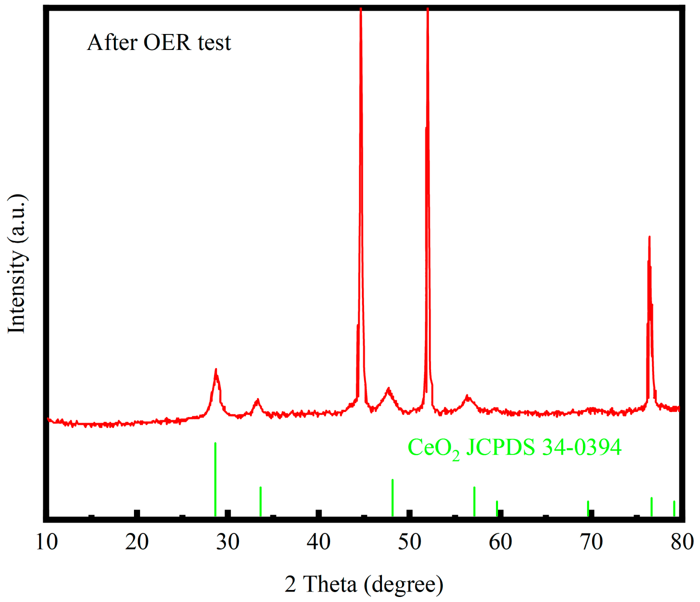

Figure S11. XRD images of Ni_3N-CeO_2/NF after HER stability test.
